# Continuous insulin administration via complex central venous catheter infusion tubing is another risk factor for blood glucose imbalance. A retrospective study

**DOI:** 10.1186/2110-5820-2-16

**Published:** 2012-06-14

**Authors:** Eric Maury, Paola Vitry, Arnauld Galbois, Hafid Ait-Oufella, Jean-Luc Baudel, Bertrand Guidet, Georges Offenstadt

**Affiliations:** 1 Service de Réanimation Médicale, Hôpital Saint-Antoine, Assistance Publique-Hôpitaux de Paris, 184 rue du faubourg Saint-Antoine, Paris, 75571, France; 2 Université Pierre et Marie Curie-Paris 6, UMR S 707, Paris, F-75012, France

**Keywords:** Hypoglycemia, Intensive care unit, Infusion tubing, Central venous catheter, Intensive insulin therapy

## Abstract

**Background:**

We assessed the potential impact of infusion tubing on blood glucose imbalance in ICU patients given intensive insulin therapy (IIT). We compared the incidence of blood glucose imbalance in patients equipped, in a nonrandomized fashion, with either conventional tubing or with a multiport infusion device.

**Methods:**

We retrospectively analyzed the nursing files of 35 patients given IIT through the distal line of a double-lumen central venous catheter. A total of 1389 hours of IIT were analyzed for occurrence of hypoglycemic events [defined as arterial blood glucose below 90 mg/dL requiring discontinuation of insulin].

**Results:**

Twenty-one hypoglycemic events were noted (density of incidence 15 for 1000 hours of ITT). In 17 of these 21 events (81%), medication had been administered during the previous hour through the line connected to the distal lumen of the catheter. Conventional tubing use was associated with a higher density of incidence of hypoglycemic events than multiport infusion device use (23 vs. 2 for 1,000 hours of IIT; rate ratio = 11.5; 95% confidence interval, 2.71–48.8; *p* < 0.001).

**Conclusions:**

The administration of on-demand medication through tubing carrying other medications can lead to the delivery of significant amounts of unscheduled products. Hypoglycaemia observed during IIT could be related to this phenomenon. The use of a multiport infusion device with a limited dead volume could limit hypoglycemia in patients on IIT.

## Background

During the past decade, there has been increasing recognition of the challenge posed by optimization of glucose management in the heterogeneous critically ill population [1-4] The method, speed and degree of glycaemic control, most importantly hypoglycaemia, are increasingly recognized and debated [5-7]. Hypoglycemia occurring during IIT has been associated with severity score, ICU length of stay [8], outcome [6], inotropic support [7] hemodialysis [9], and errors in administering insulin [10]. We present a retrospective analysis of hypoglycemic events in patients receiving continuously infused insulin via a complex central venous catheter (CVC) infusion set. We discuss concerns about the potential for untoward bolus administration of insulin and a method to limit its occurrence.

## Methods

This study was a retrospective analysis of the nursing charts of patients admitted to a 14-bed ICU affiliated with a 760-bed teaching hospital. We assessed the charts of all consecutive nondiabetic patients during a 6-month period (from 13th March to 13th September 2009) equipped with an arterial line who were receiving multiple (more than 2) intravenous therapies, including IIT through a double CVC. The Institutional Review Board of our hospital authorized this retrospective analysis. All patients or next of kin gave their consent for the data obtained during their ICU stay to be retrospectively analyzed.

**Figure 1:**
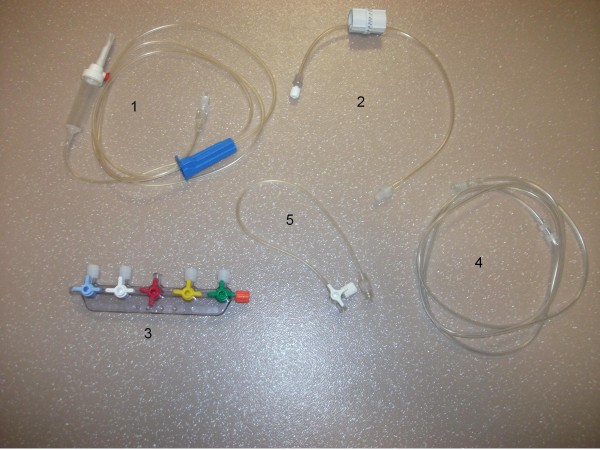
The components (numbered in the order in which they were connected) of the infusion tubing through which medications other than vasoactive agents are administered via the distal lumen of the central venous catheter: a 175-cm infusion tube [1], a 45-cm DosiFlow® [2], a 20-cm 5-way valve ramp [3], a 50-cm extension [4] and a 45-cm 1-way tube [5].

**Figure 2:**
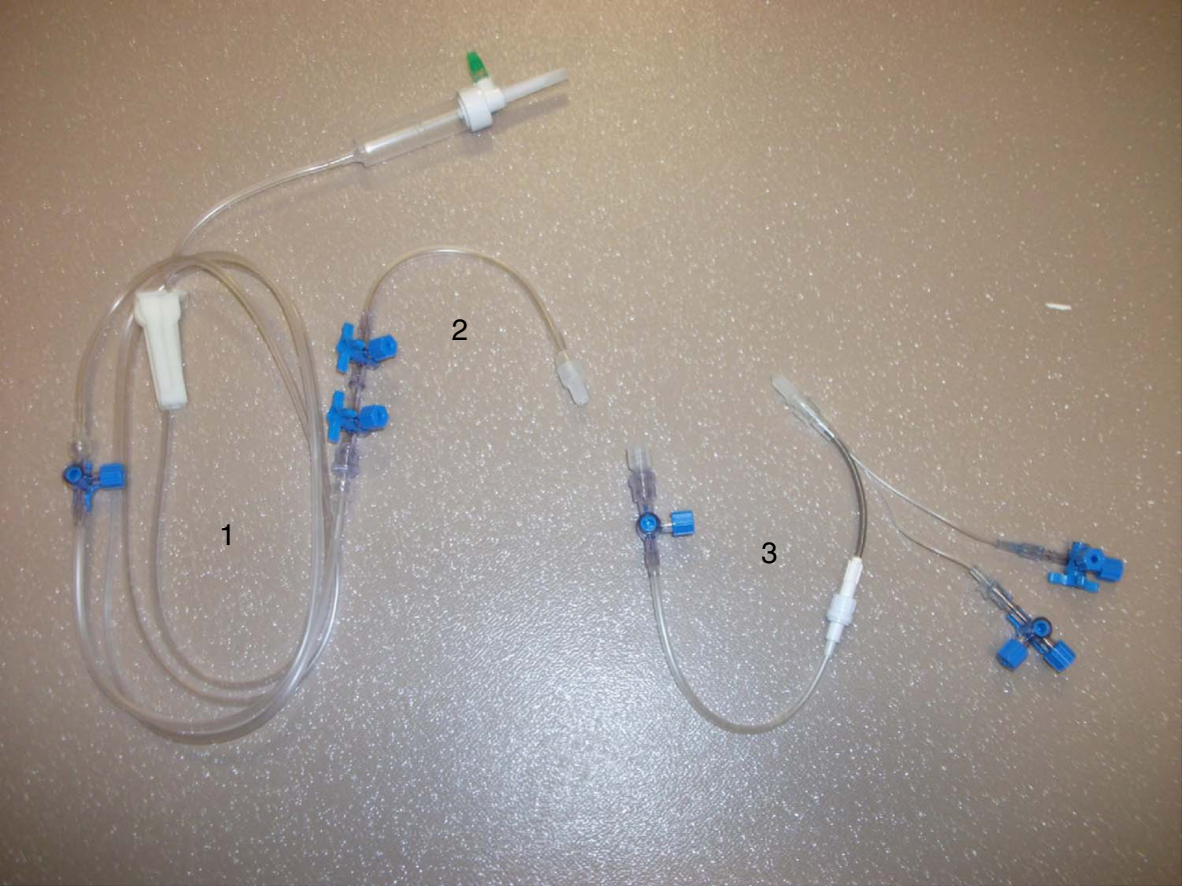
Components of the multiport access device. Infusion tubing (1), 2-way ramp (2), distal multi-access port with a 0.6 mL dead volume (3).

In our ICU, during the study period, severely ill patients receiving vasoactive agents were equipped with a double-lumen CVC, through the proximal lumen of which vasoactive agents (vasopressors and vasodilators) were delivered. All remaining medications (antibiotics, sedatives, diuretics, fluids, insulin) were administered through the distal lumen. The tubing connected to the distal lumen includes, from the infusion bag to the patient, a 175-cm infusion tube, a 45-cm DosiFlow® (Asept Inmed, Balma France), a 20-cm 5-way valve ramp (Asept Inmed), a 50-cm extension and a 45-cm 1-way tube connected to the catheter (Figure 1). This tubing set-up reflects the regular care adopted, during this period, by the nursing team of our institution. During the study period, the nursing team was offered the opportunity to use a registered device (Edelvaiss 12 + R, Doran Intl, Toussieu, France), a limited number of which were made available by the Pharmacy Department of our institution. Use of this multiport infusion device was intended to simplify the administration of multiple medications through the same catheter lumen. It offers multiport access to an infusion tube connected to the distal lumen of the CVC (Figure 2). The use of the multiport infusion device was purely a nursing decision. Trialing of the new device was contingent upon a requirement for complex tubing (more than 2 medications infused through a double-lumen CVC). To avoid severe hypoglycaemia, we used a glycaemic control protocol less stringent than the Leuven protocol [1]. Briefly, the purpose of our protocol was to start intravenous insulin when blood glucose was ≥ 150 mg/dL. Insulin infusion (50 IU of Actrapid [Novo Nordisk, Copenhagen, Denmark] in 50 mL of 0.9% sodium chloride) was started with the use of a pump (Perfusor-Space, B. Braun Medical, Boulogne, France) as follows: 2 IU/h between 150 and 179 mg/dL, 3 IU/h between 180 and 219 mg/dL, 4 IU/h between 220 and 259 mg/dL, and 4 IU as a bolus and then 6 IU/h when blood glucose was ≥ 260 mg/dL. Further insulin infusion was adjusted according to blood glucose. When blood glucose was ≤ 200 mg/dL and had decreased by ≥30 mg/dL, insulin infusion was halved. Infusion was not modified when blood glucose was between 100 and 150 mg/dL. Conversely, it was increased by 1 IU/h when blood glucose was between 151 and 199 mg/dL or by 2 IU/h when blood glucose ≥200 mg/dL. Insulin infusion was stopped when blood glucose was ≤ 100 mg/dL and 30% dextrose was given when blood glucose was ≤70 mg/dL. Insulin was restarted when blood glucose was ≥ 130 mg/dL. By comparison, in the Leuven protocol, insulin infusion was started if the blood glucose level exceeded 110 mg/dL, and the infusion was adjusted to maintain blood glucose between 80 and 110 mg/dL.

The features of hypoglycemic events while blood glucose was stabilized were analyzed. Stabilized blood glucose was defined as an insulin regimen unmodified for at least 3 hours in a patient receiving continuous enteral or parenteral nutrition. Hypoglycemic events were defined as the occurrence, in a patient previously receiving 3 IU or more of intravenous insulin hourly, of arterial blood glucose ≤ 90 mg/dL, for which insulin was stopped. Mild hypoglycemic events were defined as the impossibility of restarting insulin therapy for 2 hours; moderate hypoglycemic events required discontinuation of insulin for at least 3 hours. Blood glucose was initially measured hourly and then every 4 hours when insulin infusion was unmodified for 4 hours. Arterial blood glucose was measured using an indwelling catheter inserted for hemodynamic monitoring with the use of a hand-held blood glucose meter (Accu-Chek Performa, Roche Diagnostics France, Meylan, France). We compared patients according to the tubing system used (conventional tubing system or multiport infusion device) and assessed the incidence of hypoglycemia associated with the tubing system used.

Continuous variables are expressed as median with quartile intervals and compared using the Mann–Whitney *U* test. Categorical variables are compared using the Chi-squared or Fisher’s exact test as appropriate. The densities of hypoglycemic events for 1,000 hours of IIT were compared using the rate ratio.

## Results

**Figure 3:**
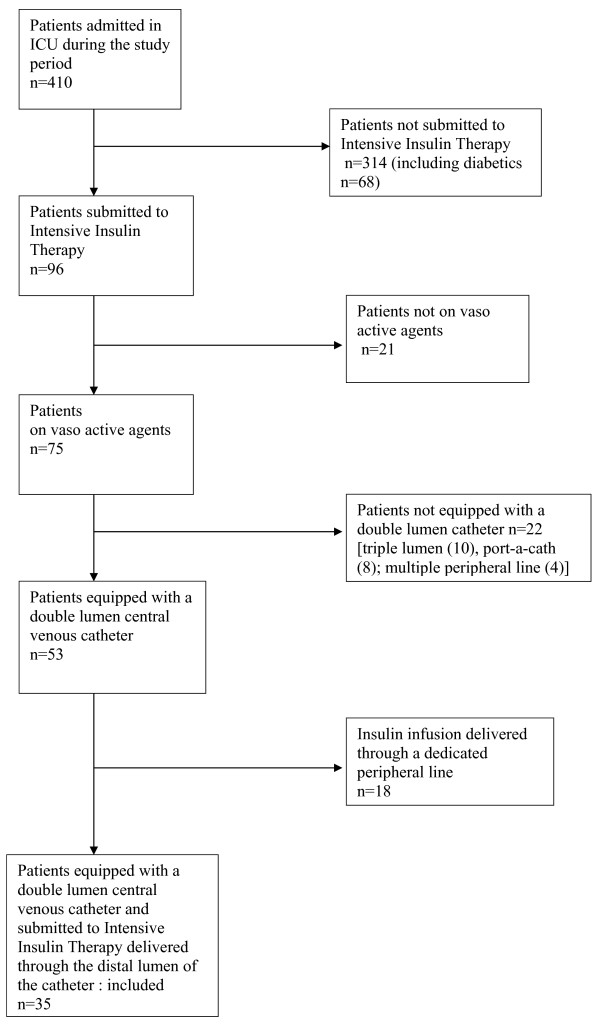
Patient screening flow chart.

During the study period, 35 of the 410 patients (23 men; age, 59 years; SAPS II, 47) admitted to our ICU were enrolled in the analysis (Figure 3; Table 1). Thirty of them received both invasive mechanical ventilation and continuous intravenous sedation. Thirty-two received vasopressors and three were given vasodilators through the proximal lumen of the CVC. The median duration of IIT was 42(extremes, 12–184 hours). A total of 1,389 hours of IIT was analyzed. The median number of glucose measurements per day was 14(range, 8–22). The number of glucose measurements per patient was similar in the two groups (13/day vs. 15/day). Mean blood glucose level did not differ between the two groups: 161 ± 36 mg/dL in the group equipped with regular tubing and 157 ± 32 mg/dL in the group equipped with the multiport device. Twenty-one hypoglycemic events were observed (incidence: 15 per 1,000 hours of IIT) as follows: 13 mild hypoglycemic events in 11 patients and 8 moderate hypoglycemic events in 7 patients. Blood glucose was always ≥ 70 mg/dL in hypoglycemic events, which did not require 30% dextrose. In 17 of these 21 hypoglycemic events (81%), the patient had been given, during the hour before the hypoglycemic event, diuretics (n = 5), antibiotics (n = 7) or a sedative bolus (n = 5) through the line connected to the distal lumen. These medications were infused in a bag of 50 or 100 mL connected to the 5-way valve ramp. The measured dead volume from the 5-way valve ramp to the patient was 12.5 mL (range 12.0–13.1), whereas the measured dead volume of the multiport infusion device was 0.6 mL (range, 0.5–0.8).

**Table 1:** Patient characteristics according to the device used to administer medications through the distal lumen of the central venous catheter

	**Regular tubing**	**Multiport infusion device**	***p***
Patients (n)	20	15	
Sex ratio: M/F	11/9	12/3	0.16
Median age (yr)	58	60	0.2
SAPS II	45	48	0.4
Mechanical ventilation	17/20	13/15	1
Days on mechanical ventilation	10 [2–25]	11 [4–32]	0.8
ICU length of stay (days)	12 [7–25]	14 [9–32]	0.8
ICU mortality n (%)	8(30)	5(33)	1
Cumulative IIT duration (h)	863	526	
IIT duration median range (h)	48 [18–184]	36 [12–92]	0.06
Mild hypoglycemic event	12	1	
Moderate hypoglycemic event	8	0	
Hypoglycemic event incidence for 1000 h of IIT	23	2	<0.001

The analysis of the hypoglycemic events showed that their incidence was significantly decreased when the multiport infusion device was used (23 vs. 2 for 1,000 hours of IIT; rate ratio = 11.5; 95% confidence interval (CI), 2.71–48.8; *p* < 0.001; Table 1). Only one mild hypoglycemic event was observed when the multiport infusion device was used. No medication infusion was noted prior to this event.

## Discussion

Hypoglycemia is the main side effect of IIT in ICU patients [3-9], although there is little evidence that hypoglycaemia results in poor outcome in critically ill patients [3,11,12].

In this study, compared with classic tubing, the multiport infusion device was associated with a huge reduction in hypoglycemic events, possibly because of its low dead space.

As recently suggested [13], when patients are given IIT, care should be taken to use appropriate methods to: 1) infuse insulin (syringe-driven pump on a central line),and 2) measure blood glucose (prefer arterial to capillary samples, use suitable measurement device). However, to our knowledge no study has assessed the putative role of infusion tubing. It also is intriguing to note that reports of large multicenter, randomized, controlled trials assessing the impact of IIT make no mention of the tubing system used [1-4,12]. The present study suggests that for patients on IIT and receiving multiple medications through complex infusion tubing, blood glucose variations may occur after interventions involving the infusion tubing, in the absence of any change in insulin infusion rate.

When using complex tubing, bolus administration of medication (sedation adjustment, antibiotic therapy, diuretics) results in the delivery of all the solutes present between the site of administration and the tip of the catheter. In the present study, the measured volume of solutes delivered to the patient in case of bolus administration through the 5-way valve ramp was 12.5 mL.

Our study does, however, have several limitations. First, it was a single-center, nonrandomized, retrospective analysis and has to be considered as a preliminary study. Second, even though the impact of the device on glycaemic control was not implicitly assessed, nurses were asked to assess the new device’s use and acceptability. We cannot therefore exclude that, when using this new device, the nursing team provided a more intensive level of care to the patient, leading to a more adequate insulin delivery. Third, in contrast to the findings of recent studies [5-7], no severe hypoglycemia was observed in the present survey. This could be due in part to our less stringent blood glucose target levels compared with those of Van den Berghe et al. [1]. Fourth, the threshold of 90 mg/dL is not a usual value used to define hypoglycemia during IIT and is neither pertinent nor useful because it has no clinical implication. In fact, we chose this value and its definition not to detect hypoglycemia but to detect glucose imbalance in a patient who was previously in a steady state. We sought to show that complex infusion tubing can modify the accuracy of medication delivery. Insulin discontinuation when blood glucose was below 90 mg/dL also could explain in part the low rate of hypoglycemia that we observed. We acknowledge that insulin interruption is not a practice adopted by all intensivists prescribing IIT worldwide, although it was part of the NICE-SUGAR protocol [3]. Fifth, the shorter median duration of IIT in the group of patients equipped with the multiport device could in part explain the difference in the incidence of hypoglycemic events. As a matter of fact, the duration of IIT has been associated with an increased incidence of hypoglycemia [8]. However, this duration difference seems unlikely to be responsible for the observed in incidence difference of hypoglycemic events.

## Conclusion

The use of a multiport infusion device with a limited dead space is associated with a lower incidence of hypoglycemic events. This is probably related to the fact that flushing of the tube contents is avoided with the multiport infusion device. Physicians should be aware of this phenomenon, which deserves further investigation.

## Competing interests

The authors declare that they have no competing interests

## Authors’ contributions

EM and PV designed the study. EM, PV and AG conducted the study. EM, AG, HAO, JLB, BG and GO drafted the manuscript. All authors read and approved the final manuscript.
